# One Merry Christmas Bell

**Published:** 1892-12-24

**Authors:** 


### The Hospital

An Institutional Journal of

**Science, Medicine, Nursing, and Philanthropy.**

*For Hospitals, Asylums, Infirmaries, Dispensaries, Medical Practitioners, Students, Nurses, and the Charitable Public.*

Vol. XIII.—No. 326.] [Dec. 24, 1892.

### One Merry Christmas Bell.

John Bull does not always take his pleasures sadly, whatever our French neighbours may say to the contrary. He fills himself a full bumper of joy at Christmas-tide, and quaffs it like a man. The first Christmas Day ushered in an eminently practical religion. We English are above all things practical: and we have good reason to be proud of it. "Peace upon earth!" That we pass by for the present, because even we ourselves have not realized it yet. But "Goodwill among men" we claim as a secured possession. The Englishman is the most tolerant of men: what is that but "goodwill"? He is the most practically benevolent: what is that but goodwill? Let us take moral stock of ourselves in this year of grace 1892; and let us not be ashamed, either of our theoretical morals, or our practical religion, or of the wholesome fruits of both.

In religion and in politics we are, we say, good-natured. This is not because we do not feel ardently, but because we have a ruling spirit of common sense. In practical benevolence we are conspicuous above all the nations. It is astonishing how much good work manages to get itself done among us in a single year, notwithstanding that we are experts in the art of grumbling. The foreigner, who reads our newspapers but is not familiar with our ways, might be excused if he should think us the most captious and the least goodnatured people on the face of the earth. The exact reverse of this would be nearer the truth. We grumble, but even whilst we are into the act of grumbling we are putting our hands in our pockets and expressing either a modified satisfaction or a hope that something better will be done next time. The Englishman admits that he has seen an end of all perfection; but then he never expected to find perfection in anything human. It comes to this, therefore, in practice, that whenever he sees honest work honestly attempted, and done reasonably well, he is entirely pleased, though he does not always say so.

In our present issue we have endeavoured to approach Christmas from many standpoints, but the one Christmas bell which we feel we ought to ring on this Christmas morn is the bell which announces a year's noble, benevolent, and scientific work done and completed at the British hospitals. In describing this work there is absolutely no need for a single word of exaggeration or philanthropic "drumbeating." We shall tell our brief story of "goodwill among men" in the simplest possible language, fully assured that we cannot convey, with all our most ardent wishes, so much as a thousandth part of the actual truth of the case. Christmas is the time for ghosts. What a magnificent army of flesh-and-blood ghosts would appear if one could suddenly make all the men, women, and children pass in array who have been cured of deadly ills at the hospitals during the last twelve months! They would constitute a fine corp of evangelists to proclaim their belief in the gospel of philanthropy. Whoever does not believe in the practical goodwill of the English people, they do. They would answer objectors as the blind man in the New Testament narrative answered those who doubted the miracle of healing: "One thing we know," they would say, "that whereas we once were at the point of death, now we are cured and well."

The man who has given some of his money to the hospitals has the best of reasons for feeling a warm glow at his heart as he pictures to himself the eight or nine hundred thousand "cured" folk who have shared in his kindness during the past year. Even figures become lively reading when every separate unit represents a living person who owes a fractional debt of gratitude to the reader. Perhaps the 874,008 out-patients at the various London hospitals last year were not among the cleverest or the most interesting of Londoners. But there is something to be said even for them. They take life with a certain easy indifference, which, though not conducive to ambitious success, yet has something to say for itself. Perhaps it is as well for some of us who are ambitious that the company of indifferents is so very large. It is not impossible that some, even of the out-patients, may be cleverer than we are, and if they would but put their best foot foremost they might leave us well behind in the race. To-day we will try to see the best side, even of the out-patients.

The in-patients are a different class. They deserve all our most tender consideration. They number 88,562. These are respectable and respected people; four or five thousand of them are children; but most of them are men and women of the "bread-winning" class. Over 51,000 of these are found in the general hospitals. They are the victims of acute diseases, such as typhoid fever, pneumonia, and the like; or of various accidents, dangerous to life or limb. More children, to the number of 6,000 in-patients and over 91,000 out-patients, are received and treated at the hospitals specially set apart for children. These hospitals are in practice as much "associations for the prevention of cruelty to children" as the noble society which goes by that special name. The sufferings among helpless children, which they annually prevent, are of themselves an amply sufficient reason for the existence of these children's hospitals. But the sufferings are insignificant compared with the cures. At the children's hospitals, the "blind receive their sight, the deaf hear, the lame walk," and almost "the dead are raised up."

There is not space to describe the work of all the special hospitals at length, but every man and woman who breathes our winter fogs and wraps himself closely against the fierce perversities of an English winter will be thankful to know that more than 45,000 of the victims of chest diseases find refuge and care at the chest hospitals every year. Perhaps the most hardly-pressed of all the five millions of London's population are the over-worked and under-fed women who work for their own livings. It will relieve the heart of every kindly woman to know that more than 40,000 of these obtain rest, refreshment, and cure in the London hospitals annually. The remaining special hospitals—for diseases of the Eye, the Ear and Throat, Cancer, the Skin, and other parts and affections of the body—treat more than 177,000 patients during the year, of which over 20,000 are serious cases, and require to be taken in as in-patients. In short, the amount of work done at our 119 hospitals during a single twelvemonth is simply incredible. How many of us are aware that 700 of the best educated and ablest physicians and surgeons in the world are giving their services to the poor people in the hospitals all the year round? Not only so, but more than 1,000 nurses of the most highly-trained class are fully employed in this service. It is a very striking history. What public service have we in this country of which all may be more justly proud? What public work can we look back upon during the past year which can give greater zest to our Christmas pleasures, or a keener appetite for our Christmas feasts? The ringing of this one Christmas bell on this Christmas morn makes sweet music in every wholesome heart.

Having gone so far, we are well prepared to look ever at the bill of costs with merry hearts; nay, the very prodigiousness of the bill makes us all the more proud and satisfied. £586,172 is the magnificent sum which of our own free will we have spent on our sick poor during the past year, and of this amount we have already taxed ourselves, or are about to tax ourselves, to the tune of £241,622. With such facts before us we feel that we cannot condescend to beg. Begging is not the note for Christmas time. Even the very "waits" disdain to play the part of beggars. They give us their music with the best of good feeling, and with proud modesty await the reward of their skill. They are never disappointed; nor can the hospitals be. From every part of our country game, flowers, fruits, and other gifts have been poured in, and every hospital ward in London is merry with dancing eyes and hearts. The cheques and the bank notes will surely follow; it is inevitable. England is still England: our hearts are where they were. However great be this work of benevolence which our fathers have handed down to us, we shall take it up proudly and resolutely; and we shall pass it on to succeeding generations greater and nobler than we found it. "Goodwill among men," above all "goodwill" to the helpless and the sick, is that practical aspect of the Christian faith which has achieved a complete victory over the heart of the English nation.

